# Double-Reinforced Fish Gelatin Composite Scaffolds for Osteochondral Substitutes

**DOI:** 10.3390/ma16051815

**Published:** 2023-02-22

**Authors:** Alin Georgian Toader, George Mihail Vlasceanu, Andrada Serafim, Adela Banciu, Mariana Ionita

**Affiliations:** 1 Advanced Polymer Materials Group, Department of Bioresources and Polymer Science, University POLITEHNICA of Bucharest, 1-7 Gheorghe Polizu Street, 011061 Bucharest, Romania; 2 Faculty of Medical Engineering, University POLITEHNICA of Bucharest, 1-7 Gheorghe Polizu Street, 011061 Bucharest, Romania

**Keywords:** fish gelatin, graphene oxide, genipin, kappa-carrageenan, composite blends, osteogenesis

## Abstract

Genipin crosslinked composite blends of fish gelatin/kappa-carrageenan (fG/κC) with different concentrations of graphene oxide (GO) for osteochondral substitutes were prepared by a simple solution-blending method. The resulting structures were examined by micro-computer tomography, swelling studies, enzymatic degradations, compressions tests, MTT, LDH, and LIVE/DEAD assays. The derived findings revealed that genipin crosslinked fG/κC blends reinforced with GO have a homogenous morphology with ideal pore dimensions of 200–500 µm for bones alternative. GO additivation with a concentration above 1.25% increased the blends’ fluid absorption. The full degradation of the blends occurs in 10 days and the gel fraction stability increases with GO concentration. The blend compression modules decrease at first until fG/κC GO3, which has the least elastic behavior, then by raising the GO concentration the blends start to regain elasticity. The MC3T3-E1 cell viability reveals less viable cells with the increase of GO concentration. The LDH together with the LIVE/DEAD assays reports a high concentration of live and healthy cells in all types of composite blends and very few dead cells at the higher GO content.

## 1. Introduction

The combination of cells and a material scaffold is traditionally the foundation upon which tissue engineering is built. Originally, scaffolds were designed to support mechanical loads while allowing cells to regenerate tissue. Synthetic polymers were the best contenders because of their ease of processing and ability to provide porosity where needed [1]. As the importance of cell-extracellular matrix (ECM) interactions and local mechanical qualities such as stiffness became clearer, the focus changed from hard-polymeric scaffolds that imitate bulk tissue mechanics, to hydrogels that replicate local cell environments [2].

However, a single material does not always suffice to yield all the desirable properties (mechanical, biochemical, and structural), and as a result, composites emerged as the most-frequent type of physical support in biomedical research. In general, polymers (proteins and/or polysaccharides) and ceramics are most often used in composite templates that mimic the native structuration of bone ECM [3]. Novel composites that contain a stimuli-responsive phase to enable physical cell stimulation are also on the rise. For example, electrical conductors or magnetic phases enable electrical or remote mechanical cell stimulation, respectively [4,5]. Ergo, carbon and metal nanoparticles can be included in artificial matrices to facilitate the attainment of these particular characteristics [6].

Proteins and polysaccharides play a variety of major tasks in their native biological systems. Bioinspired approaches usually fundament protein-polysaccharide blends that outperform individual components [7,8]. In animal tissues, ECMs, the dominant protein phase is hybridized by marginal amounts of stiffer polysaccharides, but with an outstanding implication in terms of mechanical support and cell signaling [9]. Therefore, in blend design, selecting appropriate complementary materials is crucial for producing specific structuration, hydrophilicity, surface charges, patterns, etc.

Gelatin results from the hydrolyzation of extracted collagen from skin, bone, and tendon. Among others, fish gelatin (fG) is deemed immunologically the safest, has economic and environmental benefits, and is in line with religious food restrictions (theoretically applying to 39% of the world population). Unique rheological qualities make it more useful than mammalian gelatins. Due to their biocompatibility and adjustable biodegradability, they are recommended for the fabrication of tissue substitutes spanning from epithelia to bone [10]. In skeletal tissue engineering (STE), fG itself has the capacity to initiate and sustain the development of chondrogenesis from adipose-derived stromal cells [11] and osteoblastic differentiation of human mesenchymal stem cells, even more, when micromechanical cues and supramolecular protein structuration arise [12]. The current limitations of its use derive from the weak hydrogel strength. Kappa-carrageenan (κC), a water-soluble polysaccharide hydrocolloid derived from red algae, is used to raise gelatin’s gelling and melting temperatures [13]. In addition to being a gel strengthener, its mechanical toughness, gelation characteristics, and structural resemblance to glycosaminoglycan (GAG) constituents of human bone and cartilage ECM (chondroitin-4-sulphate and dermatan sulphate) [13,14] propelled κC to become one of the most intriguing polysaccharides that stimulate the metabolic activity of hard tissue cells [15,16].

Externally triggered changes in intermolecular interactions can cause polysaccharides or proteins to gel. A common feature of both fG and κC is the profile of thermo-reversible gelation. In spite of being versatile and pliable, noncovalent hydrogel blends are less stable than chemically crosslinked networks. Taking advantage of functional groups naturally decorating the backbones of fG and κC chains, customizable double-network hydrogels can be attained through covalent crosslinking.

Above all others, primary amines can serve as covalent crosslinking points for genipin, a natural and nontoxic crosslinker, used due to the stable networks it generates from both proteins and polysaccharides. Genipin crosslinking enables the creation of hydrogels with customizable structuration based on the nature of macromolecular components in the blend [16,17].

Graphene oxide particles (GO), the oxidized form of graphene, have considerable potential in STE due to an unparalleled collection of characteristics and their compatibility with proteins that make them attractive for stimulatory scaffold development for bone regeneration [18] or cartilage substitutes [19]. In particular, GO-containing composites have shown improved osteogenic performance per se, without considering that their structure can be easily tailored to better stimulate osteogenesis [20]. GO impulses osteogenesis by its cell-friendly chemistry that can encourage its use as a coating [21] of inorganic substrates, which mostly replicate the mechanics of the native tissue but lack in terms of bioactivity, favoring cell physiology processes [22], physical stimulus originated directly (durotaxis governed by matrix embedded GO micromechanical stimuli) [19,22], adhesion-encouraging topography [23], indirectly (multi-purpose filler that can tailor artificial scaffolds towards bone ECM resembling architectures) [24], or both [20].

When investigating the impact of GO loading concentration on polymer composites for tissue engineering [25,26,27,28] or the performance of other biomedical devices [29,30], limited variations are usually surveyed. The values of GO content (cGO) are often restrained to linear strings defined by equations such as cGO_n+1_ = cGO_n_ + x (e.g., 1%, 1.5%, 2%, …, where x = 0.5) and also dismiss tackling a broad coverage of “ultra-low” [31] or “very high” (A/N) [32] concentrations counted against the mass of the reinforced phase. The main objective of this study is to investigate the influence of GO concentration in genipin crosslinked fG/κC hydrogel blends. Notably, the focus of this study was on five GO nominal concentrations extracted from an exponential curve we defined in order to cover fractional and super unitary values with the margin ratio within the range of dozens (in particular ≈ 30). We expected that upon studying highly disparate concentrations, the five-point baseline could serve as an outline ensuring elemental insight into expectations for the nominal ratio adjustments contained between one of the four narrow sequential pairs.

Moreover, the blend is designed to feature κC as both a mechanical enhancer of the network, as well as a biologically active osteomodulatory component. GO is also known to act as a supporter of osteogenesis in virtue of two grounds—cell friendly chemistry, and cell-detectable micromechanical stimuli distributed across the matrix. The approach for characterizing the doubly reinforced fG networks is oriented towards identifying bone tissue similarities with our designed formulations, and addressing the cellular and acellular behavior in vitro.

## 2. Materials and Methods

### 2.1. Materials

Cold water fish gelatin, κ-carrageenan, genipin (HPLC grade > 98%), and graphene oxide (powder, 15–20 sheets, 4–10% edge oxidized) were purchased from Sigma-Aldrich (St. Louis, MO, USA). Additionally, the reagents involved in the enzyme degradation study, collagenase from *Clostridium histolyticum*, Tris-HCl, NaN_3_, CaCl_2_, and EDTA were purchased from Sigma-Aldrich (St. Louis, MO, USA). 

### 2.2. Composite Blend Synthesis

All 6 formulations had a final volume of 100 mL at a total polymer concentration of 5% *w*/*v*, and a constant κ-carrageenan: fish gelatin weight ratio of 1:3. Graphene oxide content (cGO) was varied exponentially based on the equation:

cGO [%] = 1.25^x^, (1)

where x = ±7.5, ±3.75, and 1, and represents weight percentages (*w*/*w*) of the total polymer; the detailed rationale behind this can be found in the Supplementary Material, as well as the explicit content of each formulation in Table S1.

GO dispersions were obtained in double-distilled water (30 mL each). In brief, the exfoliation procedure was carried out using a VCX 750 ultrasonic device from Sonics & Materials, Inc. (Newton, CT, USA) provided with a Ti-6Al-4V probe tip and a 750 W processor operating at 20 kHz in a pulse/pause regime of 10/5 s tip vibrations, preset at 70% amplitude. The GO powder was added to a volume of 30 mL distilled water in a 50 mL beaker. The beaker was immersed in an ice-cold water bath, sealed after probe tip immersion in the dispersion medium, and throughout the 60 min (total pulse) procedure, the bath was supplemented three times with equal amounts of ice in order to preserve constant temperature.

**Figure 1:**
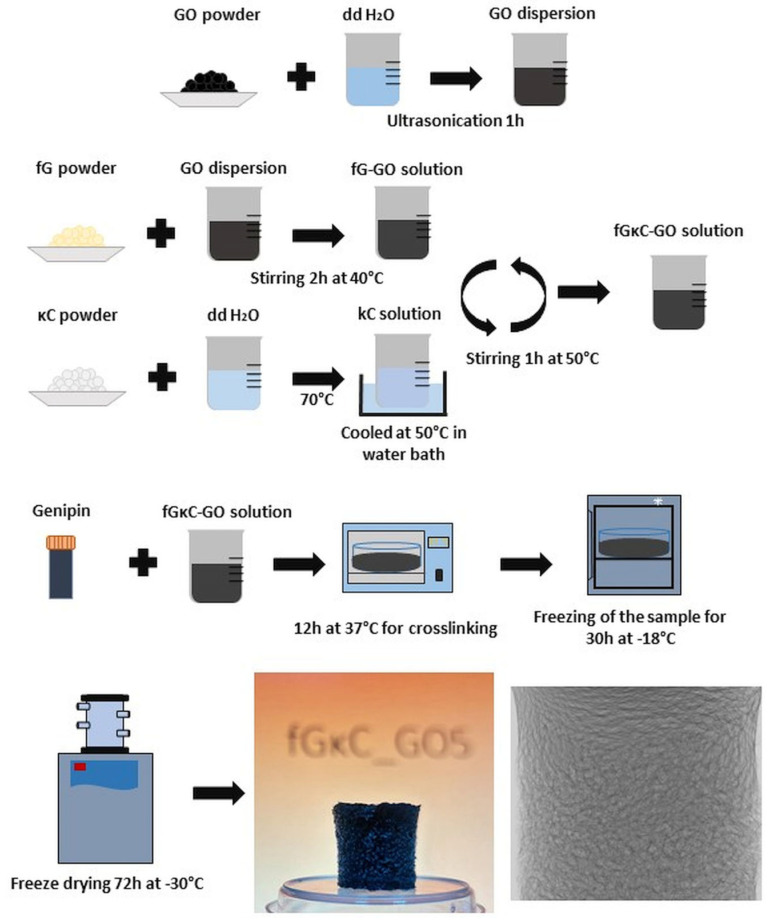
Schematic diagram of fGκC and GO reinforced fGκC formulations.

Fish gelatin was dissolved in GO dispersions under constant stirring for 2 h at 40 °C [33,34]. The κ-carrageenan solutions (70 mL each) were obtained by dissolving the powder at 70 °C until a clear solution was obtained. The κ-carrageenan solutions were cooled at 50 °C in a water bath and mixed with fish gelatin-GO solutions while thoroughly stirring for 1 h at 50 °C. The crosslinker, genipin, was added in a molar ratio of 0.1 mol/mole of fish gelatin NH_2,_ and the pairs galactopyranose and galactopyranose, according to reports in the literature [35]. Solutions were cast in Petri dishes and left undisturbed for 12 h at 37 °C in a stove for the crosslinking reaction to carry out completely. Afterward, the hydrogels were frozen (30 h, −18 °C) and freeze-dried (72 h, −30 °C, 0.01 mbar). A graphic flowchart of the batch’s synthesis is depicted in Figure 1. 

The blank composition consisting solely of the genipin crosslinked biopolymer blend is further referred to as fGκC. For the cGOs emerged for the above listed x values according to Equation (1), GO composites are denominated (from low cGOs to high cGOs) as follows: fGκC_GO1 (0.19 wt%), fGκC_GO2 (0.43 wt%), fGκC_GO3 (1.25 wt%), fGκC_GO4 (2.31 wt%), and fGκC_GO5 (5.33 wt%).

### 2.3. Methods

#### 2.3.1. Mechanical Testing

Compression tests were performed using a Brookfield CT3 texture analyzer with a compression load of 4500 g (resolution 0.5 g, according to the manufacturers’ specifications) and a TA4/1000 compression accessory. Completely hydrated samples were cut using a punching hole with a diameter of 10 mm. The height of each sample was measured with a caliper prior to testing (9 ± 1 mm). The measurements were performed in triplicate at room temperature. The samples were placed on the lower plate of the equipment and subjected to compression at a speed of 0.1 mm/s until they reached the breaking point. A stress versus strain graph was plotted using the dedicated software, and the compression modulus (E′, kPa) was computed from the slope of the linear part of the curve at 2% strain. The ultimate compression stress was also registered. Statistical analysis was performed in GraphPad Prism (version 6, San Diego, CA, USA) for Windows. The E′, kPa results are presented as mean ± standard deviation (SD) of the triplicate measurements and E’ values were compared using a one-way ANOVA and Bonferroni post-test. *p*-values < 0.05 were considered to be statistically significant.

#### 2.3.2. Micro-Computer Tomography

The high-resolution Bruker micro-computer tomograph 1272 apparatus (Kontich, Belgium) was used to carry out the micro-computer tomography (µCT) examination. The scanning was performed with a 50 kV source voltage, a 200 µA current intensity, and a 250 ms exposure time for each frame. Scanning was performed without the filter. During the scanning procedure, a 0.2° rotation step was used to rotate the samples 180 degrees. The image was created by averaging 3 frame acquisitions for each distinct slice. The image pixel size (scanning resolution) for each of the six samples was set at 7 µm. The resolution of the 2D projections was 2452 × 1640 pixels. Tomograms were reconstructed from the 2D radiography dataset using the Bruker NRecon 1.7.1.6 program (Kontich, Belgium). To visualize reconstructed tomograms, CTVox 3.3.0 r1403 (Bruker, Kontich, Belgium) and DataViewer 1.5.4.6 (Bruker, Kontich, Belgium) were employed, and CTAn 1.17.7.2 was used for sample analysis (Bruker, Kontich, Belgium). In CTAn, the tomograms were thresholded to separate the specimen walls from its pores, de-speckled to remove residual scanning artifacts, and 3D-analyzed to numerically quantify total porosity (tp), structure separation (pore dimensions), and structure thickness (wall thickness). After thresholding, the tomogram pixels were binarized (solid sample pixels were white, and pores were black). A quantitative study utilizing a 7 µm scanning resolution identified the width domains of solid walls/specific porosity by computing the object feature-size equivalent to respective white/black 3D pixels (voxels). CTAn can separate size-specific color-coded object features such as unique pore domains; after binarizing the dataset, the images were inverted to display and measure pores as solid objects and separate them by thresholded boundaries. CTVox displayed the pore tomograms inserted into the 3D solid scaffold to better understand the pore network interface and in-volume distribution, in a similar fashion to [36].

#### 2.3.3. Swelling Behavior

Swelling studies were used to determine the synthesized scaffolds’ ability to absorb aqueous fluid. Pre-weighted dry scaffolds were submerged in 5 mL phosphate buffer solution (PBS, pH 7.4) at room temperature. At regular intervals, swollen samples were removed from the incubation media, and weighed after wiping off the extra water with filter paper. The swelling degree (SD) was determined using Equation (2) [37]:

SD (%) = (*W*_d,t_ − *W*_0_)/*W*_0_ × 100 (2)

where *W*_d,t_ is the total weight of the swollen sample at time t and *W*_0_ is the dry weight of the scaffold prior to incubation.

#### 2.3.4. Enzyme Degradation Study

The reported enzymatic degradation was based on a previously described method [38]. Equal size (15 mg) freeze-dried specimens were incubated in a 3 mL Tris-HCl (0.1 M, pH 7.4) buffer solution enriched with 0.005% (*w*/*v*) NaN_3_ and 5 mM CaCl_2_ for 1 h, and then 1 mL of collagenase solution (15 µg/mL). The test was carried out at 37 °C in triplicate for each formulation and then averaged. Sample degradation was stopped by chemical and thermal inactivation after the addition of 0.5 mL of 0.25 M ice-cold EDTA solution and cooling on the ice bath. After collagenase inactivation, samples were washed with ice-cold Tris-HCl and distilled water. Gel fraction (GF) was determined after sample-drying in air at 37 °C by employing the following equation [39]:

GF (%) = (1 − W_d,t_/W_0_) × 100 (3)

where W_d,t_ is the weight of the dried sample, at time t of degradation, and W_0_ is the initial weight of the sample.

#### 2.3.5. In Vitro Biocompatibility

The MTT test was performed using an extract of the samples obtained in accordance with ISO 10993-12:2021(E) guidelines for sample preparation in the biological evaluation of medical devices [40]. In order to obtain the liquid extracts, materials were maintained in MEM α (M8042, Sigma, St. Louis, MO, USA) supplemented with 10% Fetal Bovine Serum (FBS, F7524, Sigma, St. Louis, MO, USA), 1% L-glutamine and 1% Penicillin-Streptomycin (P4333, Sigma) for 72 ± 2 h at 37 °C, 5% CO_2_. The MC3T3-E1 cells (ATCC CRL-2593) were seeded at a density of 1 × 10^5^ cells/mL in 96-well tissue culture plates (VWR, Radnor, PA, USA) and incubated for 24 h in standard culture conditions. The extracts were tested at different concentrations (100%, 50%, 25%, 12.5%), and after 24 h the sample extract was removed and MTT (1 mg/mL) was added [41]. After 2 h of incubation, DMSO (dimethyl sulfoxide) was used to solubilize the formazan crystals and the optical density (O.D.) was determined at 570 nm and 650 nm (reference wavelength) with the DS-11 FX+ Spectrophotometer (DeNovix).

MC3T3-E1 cells were cultured in the same culture medium and conditions as described, then seeded at the same density on glass coverslips and maintained for 24 h in a culture medium. After incubating the cells with scaffolds’ extracts for an additional 24 h at 37 °C, 5% CO_2_, the cellular viability was assessed with calcein AM (LIVE/DEAD Viability/Cytotoxicity Kit for mammalian cells, L3224, Life Technologies, Waltham, MA, USA) and Propidium Iodide. The samples were rinsed with Phosphate Buffered Saline (PBS, P3813, Sigma-Aldrich) and were incubated for 40 min at room temperature with 2 µM Calcein-AM and 1 µg/mL Propidium Iodide. After incubation, 1 mL PBS was added, and the viability was determined by fluorescence microscopy with a Zeiss LSM 880 confocal system (Zeiss, Oberkochen, Germany) with 488 and 514 nm lasers, and images were processed with the ZEN 2.3 software (Zeiss, Oberkochen, Germany).

For the LDH assay (CyQuant LDH Cytotoxicity Assay kit, Invitrogen), cells were maintained for 24 h in extracts and after the incubation, the medium was collected and evaluated according to the manufacturer’s instructions [41]. LDH activity was measured using 490 nm and 680 nm absorbance with the DS-11 FX+ Spectrophotometer (DeNovix).

## 3. Results and Discussion

The six materials which individualize only by means of cGO were characterized immediately post-synthesis: i. in the hydrated state from a mechanical point of view, ii. after freeze-drying in terms of morphological specificities via micro-computer tomography, and iii. focusing on in vitro behavior in aqueous media by addressing swellability and gel fraction against collagenase digestion. The last segment relies on addressing standard cytocompatibility assays meant to investigate possible correlations occurring between dissimilar degrees of GO reinforcement and cell fate when in contact with fGκC networks. Above all else, collected data from material analyses were gradually correlated to a maximum in a logic cascade aimed at painting a comprehensive picture of how particular cGOs alter fGκC hydrogels in various respects. 

### 3.1. Causal Links between Composition-Mechanics-Architecture Fluidity 

For bone tissue engineering, a rigid/elastic equilibrium could be ideal for the in vivo outcome of the material since it will need to have the strength to support bone formation while also withstanding the continuous mechanical load. Compression testing was performed in order to address rigidity variations occurring alongside GO fraction increase within the composites. 

**Figure 2:**
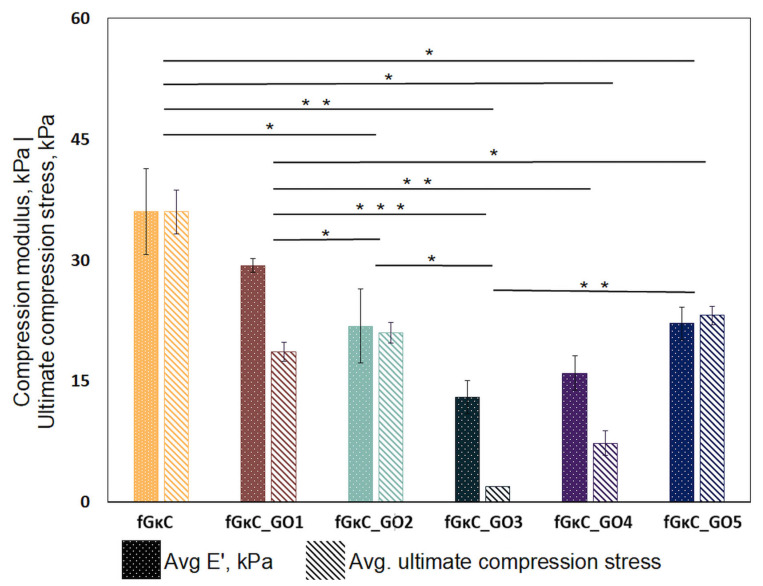
Compression behavior of synthesized materials. *** *p* < 0.0005, ** *p* < 0.005 and * *p* < 0.05 indicate statistical difference.

The outturn of mechanical stress loading revealed an interesting behavior when materials are comparatively assessed vis a vis cGOs (Figure 2). The network restructuration induced by the seemingly minor cGO difference reflected onto the elasticity of the composite gels. This was enabled by multiple interactions occurring after the secondary reinforcement with GO, between the carbon nanoparticles and polymer blend matrix. As expected, compared to the pristine blend, rigidity was increased during GO embedding in the networks, but after a certain threshold ratio, the composites partly regained their elasticity.

In brief, with respect to fGκC, formulations enriched with subunitary cGOs started to exhibit a decline in elasticity (of up to 38 in the case of fGκC_GO2). The intermediary composite, fGκC_GO3, features the least-elastic behavior, with a compression modulus of almost 36% of the controls. 

Interestingly, the formulation starts to regain elasticity when higher concentrations of GO are used. However, in recent papers, it has been brought to light that dispersed polymer solutions and restacked graphene oxide sheets tend to reorientate in particular layouts mainly according to the GO concentration (and to a less extent as a result of some rheological characteristic of the matrix); namely, stiffness might occur for low GO concentrations since the stacking carbonaceous layers are free to distribute in random planes of orientation, thus lowering the magnitude of elasticity, whilse superior densities of GO particles per volume unit contribute to a more ordered arrangement of particles (as the shorter distances between GO entities facilitate a chained reaction self-arrangement—preferentially along one axis) [42,43].

Following the significance of variations occurring in compression moduli vs. reinforcement degree, pivotal changes occur between the fGκC and minor-end cGO. With respect to fGκC_GO3/4/5, the significance of the mechanics of fGκC and fGκC_GO1 indicates that the increase in cGO from fractional to plenary is an influencing determinant of non-linear compression performance that could be widely overlooked in the composite science literature. 

**Figure 3:**
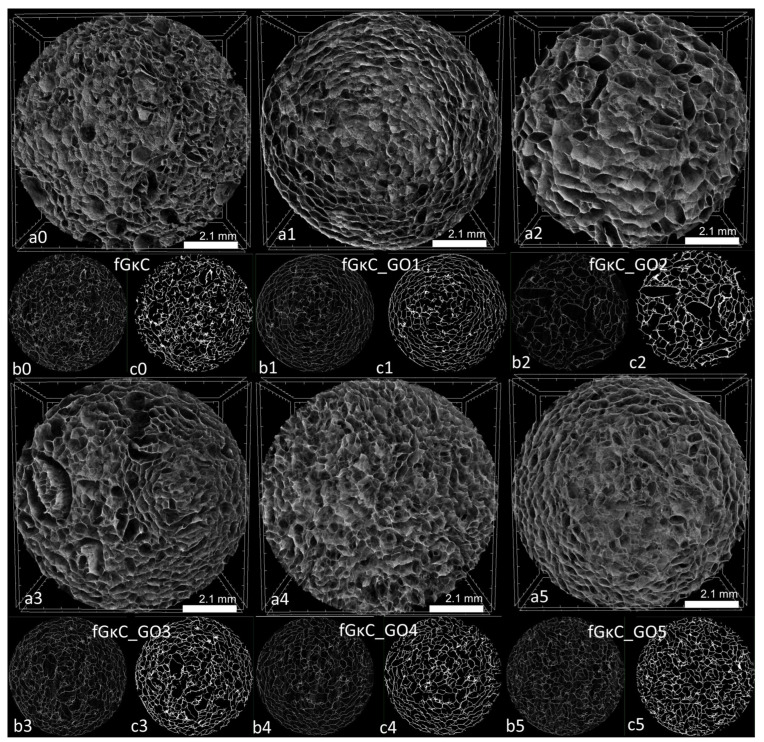
Cross-sectional captures of reconstructed tomograms illustrated in CTVox (**a0**–**a5**) and relevant slices exported from CTAn before (**b0**–**b5**) and after (**c0**–**c5**) the binarization step.

**Figure 4:**
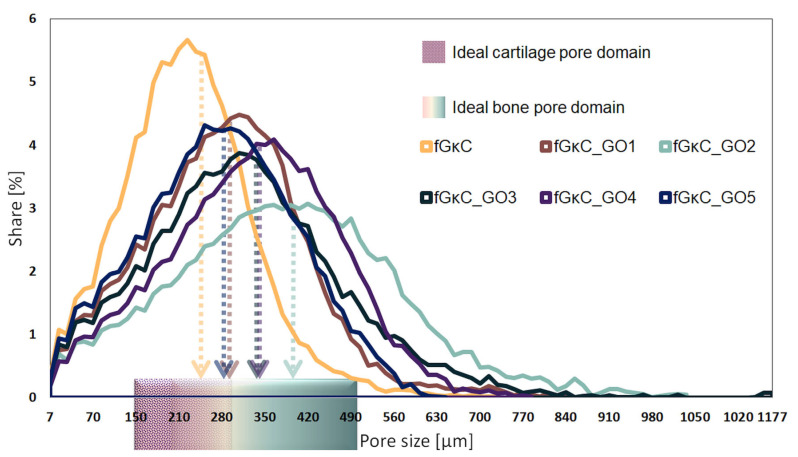
Superimposed pore size distribution of the synthesized materials against pore domains agreed [44,45] to better support cartilage and bone regeneration.

This behavior, whereby the profile of elasticity follows a concave curve, could influence solid phase organization vs. pore development in a similar way. The porosity and solid phase morphology of the freeze-dried hydrogels were studied by means of computerized tomography analysis, and qualitative (Figure 3) and quantitative (Figure 4) data are reported. 

Cross-sectional views of the scanned specimens differ marginally in terms of solid wall features and more with respect to pores, as validated by pore size analysis. As expected, there seems to be a correspondence between the elasticity of the material and the share of smaller pores that emerged within the scaffolds: the more elastic compositions feature larger fractions of small pores, which might be due to the inertia of the materials during the freezing process, limiting ice crystals augmentation. Conversely, the most rigid materials favored the solid phase separation and the patterning of a wide share of large pores. 

In CTAn, the incidence of pores on predefined size domains was calculated, depending on the scanning resolution for equally sized samples from the six lyophilized hydrogels. To attain these results, a thresholding (binarization) protocol and a scanning artifact reduction step were implemented to define with precision the solid sample (in white pixels) against the pore network (black pixels); the conversion is illustrated in (b) and (c) subsets of Figure 3. The distinct quantification of the two color-pixel domains enabled the conversion of various physical features of the specimens in common units of measurement useful in discussing the suitability of the materials in osteochondral tissue substitutes conversion. Their distribution is illustrated in Figure 4, being charted against domains consecrated in the literature as suitable for the regeneration of bone (200–500 µm) [44] and cartilaginous (150–300 µm) [45] tissues. Additionally, from a quantitative point of view, the total porosity of the samples and the incidence of pores in the areas of interest were determined, detailed in Table 1. 

The addition of GO, even in small proportions compared to the total polymer mass, factored in changes in terms of the formation of pores and less with respect to solid phase proportions. Table 1 lists the weighted average values of the mean pore (marked against the relevant tissue domains in Figure 4) and wall size; despite the fact that mean wall thicknesses for all materials are very close, important variations are, in general, associated with a predisposition of the material to form larger pores and, at the same time, to expand the domain in which they are found. This phenomenon is noticeable for samples fGκC_GO1, fGκC_GO2; marginally for fGκC_GO3 and fGκC_GO4 since they exhibit very similar features, while in the case of fGκC_GO5, the range narrows abruptly and the median drops again. The narrowest distribution occurs in the case of fGκC, for which the incidence of pores in the 150–300 µm range is higher, 40.5% (Table 1), however larger and more “flat” distributions of fGκC_GO1, fGκC_GO4, and fGκC_GO5 encompass more than 70% of total porosity within 200–500 µm which might favor bone tissue formation. 

One of our interest in studying the materials revolved also around the distributions of small pores (<50 µm) critical for the supply of nutrients for the cells and the drain of residues. Further processed images depicting the interconnected nature of the pores, as well as their color-coded size distribution, are available in Figure S1 in Supplementary Material.

It seems that the most elastic compositions (fGκC and fGκC_GO1, and the ones on the opposite extremity, fGκC_GO4 and fGκC_GO5) are the most inclined to facilitate the formation of a rather enlarged ratio of pore domains suitable for cartilage and bone regeneration. This obvious interdependency can be attributed to the significant compression modulus changes (Figure 2). 

A very common approach in the fabrication of scaffolds for the complex interface between the two types of tissue involves the employment of different materials in unidirectional anisotropic unitary objects that feature cartilage/bone specific characteristics [46]. The bilayered (but also tri- or multicomponent-layered [47]) scaffolds perform remarkably but factor in the drawback of unstable merging as a result of the chemical discrepancy between the functionally graded phases. However, the six formulations could be easily and safely paired in order to construct multilayered structures coalescing into mechanically firm and biomimetically anisotropic substituents according to the anatomy of each skeletal segment, gradually optimizing pore size among pair layers in order to mimic the cartilage vs. cortical bone and cortical vs. cancellous bone density. 

Additionally, both tomograms (subsets a) and individual reconstructed slices (subsets b) of Figure 3 show relatively balanced pore size variations in cross-sections, suggesting that although among the samples some noteworthy differences occur, the networks are robust and uniformly structured in volume. This encourages the idea of developing more accurate native-tissue multi-layered scaffolds based on fG/κC with GO reinforcing gradients. 

Alas, mechanical properties of the scaffolds might not be suitable in terms of mimicking the mechanics of bone tissue immediately after implantation. However, the four most flexible compositions fit within the range of Young’s modulus measured for natural ECMs surrounding chondrocytes and osteoblasts [48], affirming the potency of these compositions to provide familiar solidity for de novo tissue formation. 

### 3.2. Impact of Mechanics and Architecture on In Vitro Performance in Simulated Media

Scaffold architecture directly impacted the water absorption capacity and network degradation in enzyme-rich media. The formulations richest in GO and with the utmost balanced architectural features exhibited the highest swelling degree as the water was able to infiltrate through micro- and macro-channels within a network featuring significantly larger H bonding interactions as a result of GO presence. The pore patterning mediated by the rigid/elastic balance of the network is also reflected in the profile of enzyme degradation in the sense that their durability can be diminished by the presence of larger pores separating rigid walls, more susceptible to cleavage.

**Figure 5:**
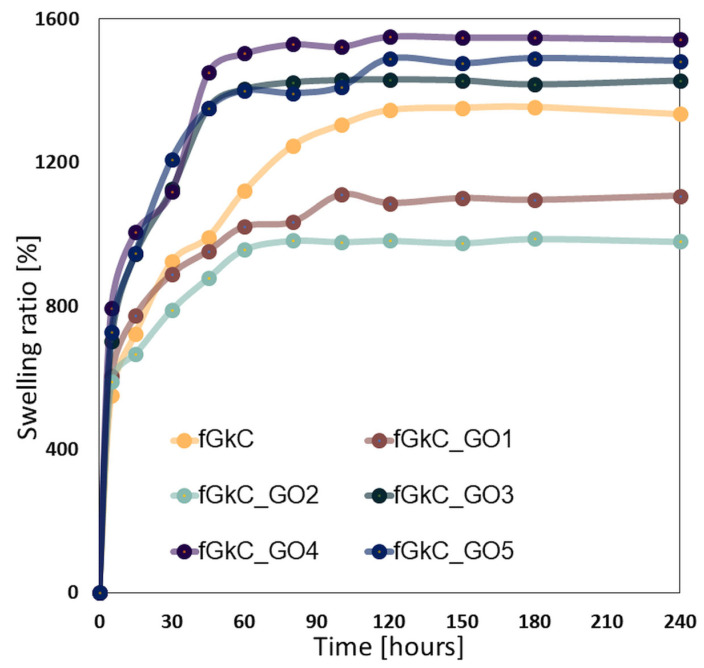
The swelling profile of the 6 formulations based on fish gelatin and k-carrageenan reinforced with graphene oxide.

The transfer of nutrients and metabolites to the cells populating a scaffold, as well as the absorption of bodily fluids are both significantly influenced by swelling capacity. The swelling behavior of the fGκC scaffolds underlies the super-porous structure of the hydrogels. Because pores are interconnected and operate as a capillary system, materials exhibit a rapid fluid uptake in the first minutes of immersion. All scaffolds, as depicted in Figure 5, establish equilibrium in less than two hours and absorb 8 to 15 times their initial weights.

These findings agree with µCT images that showed increased pore interconnectivity under the impact of GO additivation and increased fluid absorption for scaffolds with GO concentrations greater than 1.25%. This phenomenon is caused by the GO interaction with the fG/κC blend enabling the patterning of a pore network that tends to contain more pores in the lower domains, and perhaps micro-channels under the detection limit of the scanning equipment used to depict sample morphology; ergo, fluid infiltration was enhanced by the more uniform pore size (Figure 3, Table 1, Supplementary Material—Figure S1). 

On the other hand, as demonstrated by µCT, the lowest quantity of GO dispersed within the polymer matrix resulted in a decreased fluid uptake because of a reduction in pore diameter and low pore interconnectivity, coupled with higher elastic properties which first delay then permanently constrain the network expansion. This multi-sourced behavior is easier to track in fGκC_GO2, which in spite of not being the most elastic (similar to fGκC_GO5) has the most right-skewed pore distribution. 

**Figure 6:**
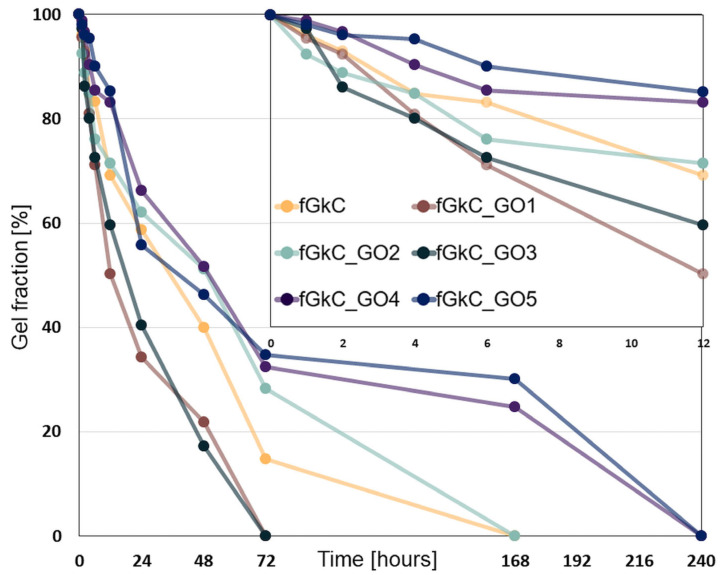
Gel fraction of the 6 formulations based on fish gelatin and k-carrageenan reinforced with graphene oxide after various times of incubation in collagenase solution.

The investigation of the stability of the materials in simulated complex physiological environments was carried out at 1/2/4/6/12/24 h, 2/3/7 and 10 days. The fastest degradation occurs in the first 6–12 h for most formulations, except for fGκC_GO4 and fGκC_GO5, where the mass loss does not exceed 20% of the initial weight of the specimens (Figure 6, inset depicting degradation kinetics in the first 12 h). This steep loss is most likely due to the removal from the network of fish gelatin chains insufficiently fixed by genipin. The improved stability of the fGκC_GO4 and fGκC_GO5 compositions can be attributed to the high concentration of graphene oxide, which in the hybrid network, due to the functional groups containing oxygen, ensures the formation of additional hydrogen bonds, interactions that contribute to the densification of the network.

The least elastic material exhibited the steepest degradation profile, and low cGO, apart from increasing the rigidity of the network, facilitated degradation (fGκC_GO1) probably via the double impact of irregular particle arrangement as disorganized domains act as weak points in the isotropy of a network. One feature that could prolong the stability of these structures is their higher elasticity (compared to fGκC_GO3 and prior) which can act as an unexpected physical adjuvant against digestion. In addition, upper cGO can also act as a barrier that slows down the collagenase diffusion in the entire polymer network and thus decelerates the degradation process [49]. The geometric feature of GO sheets and the highly impermeable character of this type of particles might impede fast collagenase diffusion, hindering vulnerable fG molecular domains, thus prolonging the functional life of fGκC composites in the wet state.

For times longer than 3 days of collagenase digestion, the degradation profile of still-withstanding formulations can also be correlated with the following: i. the materials morphological outlines, since their stability can be diminished by the presence of larger pores (fGκC_GO2) and improved as their average lowers, and ii. the scarcity (by comparison) of non-covalent interactions when cGO > 1.25 wt%. At 7 days, in the compositions fGκC_GO4 and fGκC_GO5, the gel fraction is at values comparable to the initial carrageenan fraction, and at 10 days all samples are 100% degraded.

### 3.3. In Vitro Cytocompatibility

**Figure 7:**
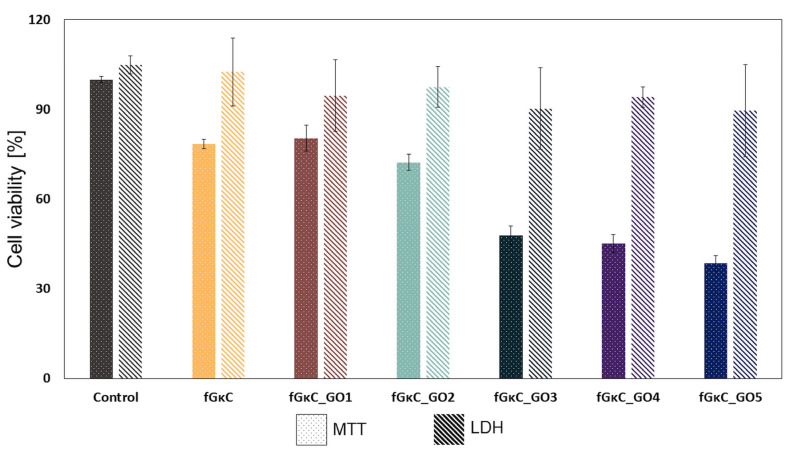
MC3T3-E1 cells viability after the exposure for 24 h to 12.5% extract of the fGκC, fGκC_GO1, fGκC_GO2, fGκC_GO3, fGκC_GO4 and fGκC_GO5 composite blends with 0, 0.009, 0.022, 0.063, 0.115 and 0.267 mg of GO.

To assess the impact of the blends on cell growth and quantify cell viability and proliferation, an MTT assay was performed on MC3T3-E1 cells after 24 h of exposure to a 12.5% extract of the fGκC, fGκC_GO1, fGκC_GO2, fGκC_GO3, fGκC_GO4, and fGκC_GO5 composite blends. The results of this assay are recorded and presented in Figure 7. The data suggest that the cell proliferation rate was positively influenced by the lowest GO content in the materials’ composition. A slight decrease in cell viability and proliferation related to the control was noticed after 24 h of exposure of the synthesized materials, and is becoming more pronounced as the quantity of GO increases [50].

More specifically, a reduced profile of cell proliferation was observed for the formulations with 0.063, 0.115, and 0.267 mg GO when compared with both pure polymer and fGκC_GO1, fGκC_GO2 composite formulation, indicating that the GO toxicity is related to its concentration within the synthesized material [51]. 

**Figure 8:**
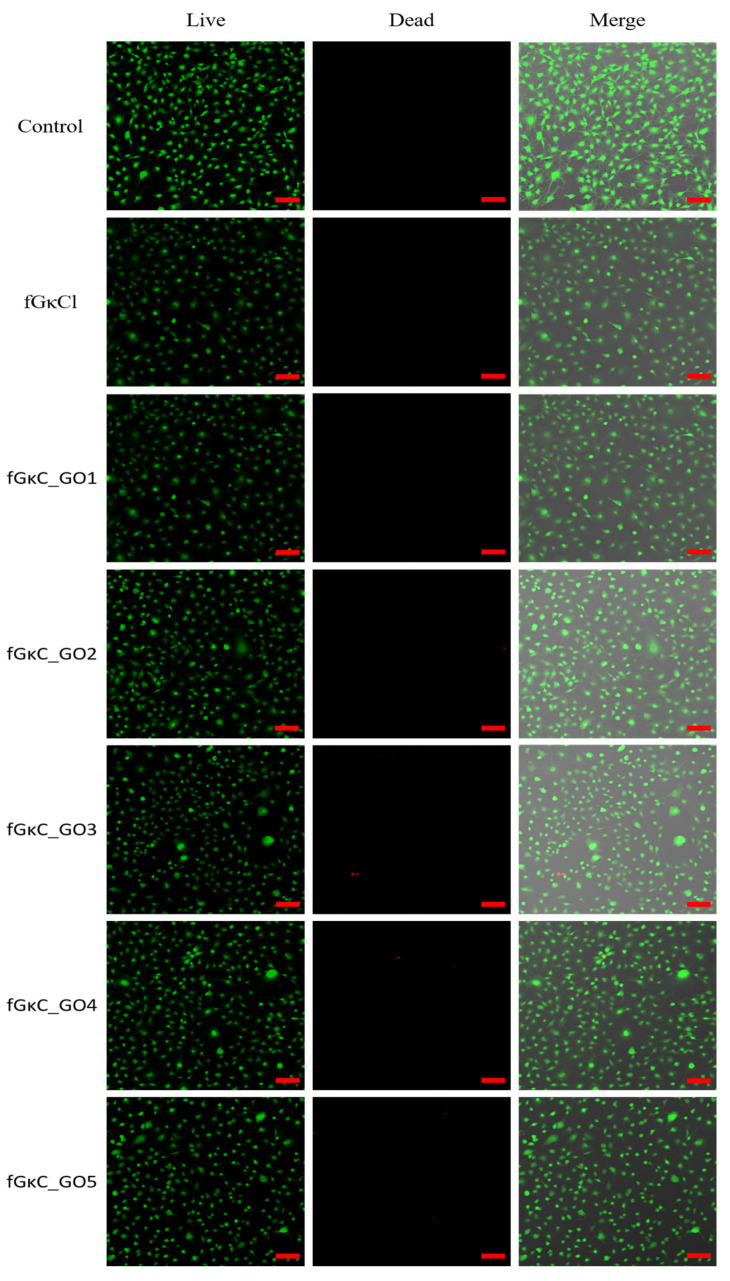
LIVE/DEAD cell viability assay. MC3T3-E1 cell viability after the exposure for 24 h to 12.5% extract of the fGκC, fGκC_GO1, fGκC_GO2, fGκC_GO3, fGκC_GO4 and fGκC_GO5 composite blends. The scale bar represents 100 µm.

In addition to the MTT assay, an LDH assay was also conducted to compare the results after 24 h of exposure. It is important to note that the results from MTT and LDH assays can sometimes differ, as has been previously reported in various studies [52,53]. For instance in the study of Jo et al. [54], in the MTT assay, these discrepancies can be caused by non-specific interactions with the tetrazolium salts. Regarding the LDH assay, the results indicated that the materials did not induce significant cytotoxicity toward the cells [50]. The low level of cytotoxicity observed after 24 h of culture was further confirmed by the LIVE/DEAD assay results (Figure 8), which showed a strong positive ratio between the live (green) and dead (red) cells.

The variability between the results obtained from quantitative assays highlights the importance of conducting complementary qualitative tests to gain a more comprehensive understanding of cellular responses. These qualitative tests, such as the Live/Dead assay, provide valuable insights into cellular morphology, membrane permeability, and enzymatic activity, in addition to enabling a more nuanced assessment of cellular response. By incorporating both quantitative and qualitative measures, a more complete picture of cellular behavior can be obtained, allowing for a more informed evaluation of the impact of different blends on cell growth. Figure 8 indicates a high concentration of green-stained cells for all the analyzed composite blends while the number of red-stained cells that represent the dead cells appears in small numbers on all the composite blends. Several studies indicated that the addition of GO in the composition of the materials generates an increase in cytotoxicity, while others reported that GO has a positive effect on cell viability, or, it was also found that cell behaviors rely on the GO concentration [55,56]. Our experiment suggests that fGκC_GO formulations favor cell viability, but to a lower extent cell proliferation when 0.063, 0.115, and 0.267 mg of GO are added to the polymer blend. Superior cell viability is, therefore, associated with higher pore shares of the samples [57] and more specific to a balanced pore size distribution proper for both cell infiltration and cell favorable metabolites exchange. In this context, the properties of fGκC_GO composite formulations in terms of cell viability and proliferation coupled with the aforementioned features related to mechanical and morphological features could favor osteochondral tissue engineering applications.

## 4. Conclusions

In this paper, we highlighted a causality between morphology-stability in vitro behavior and the stiffness of fGκC hydrogels reinforced with exponentially varied GO fractions. The GO-induced network restructuration affected composite gel elasticity, which we argue further dictated pore patterning and solid phase structuration. This was possible by GO-biopolymer noncovalent interactions occurring after the secondary reinforcement with GO. 

Mechanical characterization showed that GO addition first enhanced the rigidity of the composite compared to the pristine mix, but after a threshold ratio, the composites partly regained their elasticity and a significant non-linear correlation with cGO was highlighted. The repercussions on morphological features were studied using microCT. Due to individual elastic inertia of the composites, the differences in pore domains among the six formulations are traceable and also attributable to the acellular in vitro assessment of their wet-state behavior.

The most rigid materials supported solid phase separation and the outlining of a share of larger pores, whereas the most elastic formulations, due to material resistance, prevented their augmentation, resulting in either a sharper or more uniform size distribution. Additionally, the study found that the stability of the materials in simulated physiological environments was influenced by the concentration of GO. The samples with GO concentrations greater than 1.25% exhibited more micro-channels that increased pore interconnectivity and fluid absorption. The scaffold architecture affects water absorption and network-breakdown enzyme-rich media. The formulations with the most GO and balanced architectural features swelled the most because water could penetrate through micro- and macro-channels in a network with much greater H bonding contacts due to GO presence. The stiff/elastic balance of the network determines pore patterning, which affects enzyme degradation by making bigger pores, thus separating rigid walls more prone to cleavage.

The MTT assay showed that composite blends had fewer viable cells than the control, decreasing with GO concentration. LDH and LIVE/DEAD assays showed all composite mixes had good cell viability. High cGO composites show dead cells. DNA damage by GO molecule growth causes cell death. The variability between the results of the assays highlights the importance of conducting complementary tests for a comprehensive understanding of cellular responses. The study suggests that fGκC_GO formulations favor cell viability, but to a lesser extent cell proliferation when a certain amount of GO is added.

These findings showed how GO affects fGκC hydrogel blends. We found non-linear mechanical property fluctuations in these hydrogels that cause a ripple effect in their geometry and in vitro behavior by comparing GO concentrations. Additionally, based on these observations, further studies could focus on narrow customized GO reinforcement concentrations for more specific outcomes.

## Figures and Tables

**Table 1:** Total porosity and key pore features of the synthesized materials.

Sample	fGκC	fGκC_GO1	fGκC_GO2	fGκC_GO3	fGκC_GO4	fGκC_GO5
Total porosity [%]	86.9	88.2	88.8	88.5	88.9	87.7
Pores 150–300 µm	40.5	24.5	14.8	21.3	17.9	25.9
Pores 200–500 µm	60.67	71.3	59.2	66.1	72.0	70.2
Mean pore size [µm]	239	299	398	338	340	286
Mean wall size [µm]	28	27	31	28	27	31

## Data Availability

Not applicable.

## Supplementary Materials

The following supporting information can be downloaded at: https://www.mdpi.com/article/10.3390/ma16051815/s1, Table S1: Specific content of the protein/polysaccharide blend composites; Figure S1: Captures of reconstructed tomograms illustrated in CTVox (color figures) coupling the reconstructed pores as objects over the porous structures of the samples and representations of the intersections of the XY/XZ/ZY planes extracted from DataViewer (black and white subdivisions).
