# Supplementary material for: Double-Reinforced Fish Gelatin Composite Scaffolds for Osteochondral Substitutes
Source: Materials (Basel). 2023 Feb 22;16(5):1815. doi: 10.3390/ma16051815 (PMC10003955; doi:10.3390/ma16051815)
Supplement: Supplementary file 1 [file materials-16-01815-s001.zip › materials-2132109-supplementary.pdf]

materials

Supplementary material

Double-Reinforced Fish Gelatin Composite Scaffolds for Osteochondral Substitutes

Equation (1) of the main body of the present paper was proposed by the authors as the source of a string with numerical values ranging from marginal to large, with a moderate exponential growth. The variable was offered 5 denominations: -7.5, -3.75, +1, +3.75, +7.5 which determined the following cGO concentrations: 0.19, 0.43, 1.25, 2.31, 5.33 wt % (with Table 1 summarizing material composition of the 6 quaternary formulations). The values were considered based also on a prerequisite that ratio of the highest/the lowest concentration is the domain of dozens, but without surpassing a threshold of viscosity which could impede the homogenization of the polymer phase with the filler. It should be noted that this study concentrated on 5 GO nominal ratios that were taken from an exponential curve that we constructed in order to encompass fractional and superunitary values. The fringe values ratio (max/min) is equal to 29.66, larger than in any other singular paper the authors encountered. After examining very dissimilar concentrations, we argue that the 5 points baseline may provide a framework predicting the outcome characteristics of a composite whereby the nominal ratio adjustments is contained between-a-pair.

Table S1. Specific content of the protein/polysaccharide blend composites.

Sample	Fish gelatin [g]	κ-carrageenan [g]	Graphene oxide [g]	Genipin [mg]
fGκC	3.75	1.25	0	0.03
fGκC_GO1	3.75	1.25	0.009	0.03
fGκC_GO2	3.75	1.25	0.022	0.03
fGκC_GO3	3.75	1.25	0.063	0.03
fGκC_GO4	3.75	1.25	0.115	0.03
fGκC_GO5	3.75	1.25	0.267	0.03

Through the computerized tomography analysis, the porosity of the lyophilized samples obtained from the 6 formulations was highlighted, as well as its interconnected nature. The co-visualization (Figure 1) of the tomograms of the objects and the pores reconstructed as objects and illustrated in color codes according to their diameter provides an overview of the way in which the pores of different sizes are distributed in the sample volume. In particular, pores under 50 μm in size have a homogeneous distribution in the samples, constituting a complex network, uniformly structured in volume, through which the supply of essential nutrients for the cells can be ensured. The interconnectivity of the pores, as well as their variable sizes depending on the GO content of the materials, can be better observed in the black and white captures from the DataViewer software (Figure 1, secondary images).

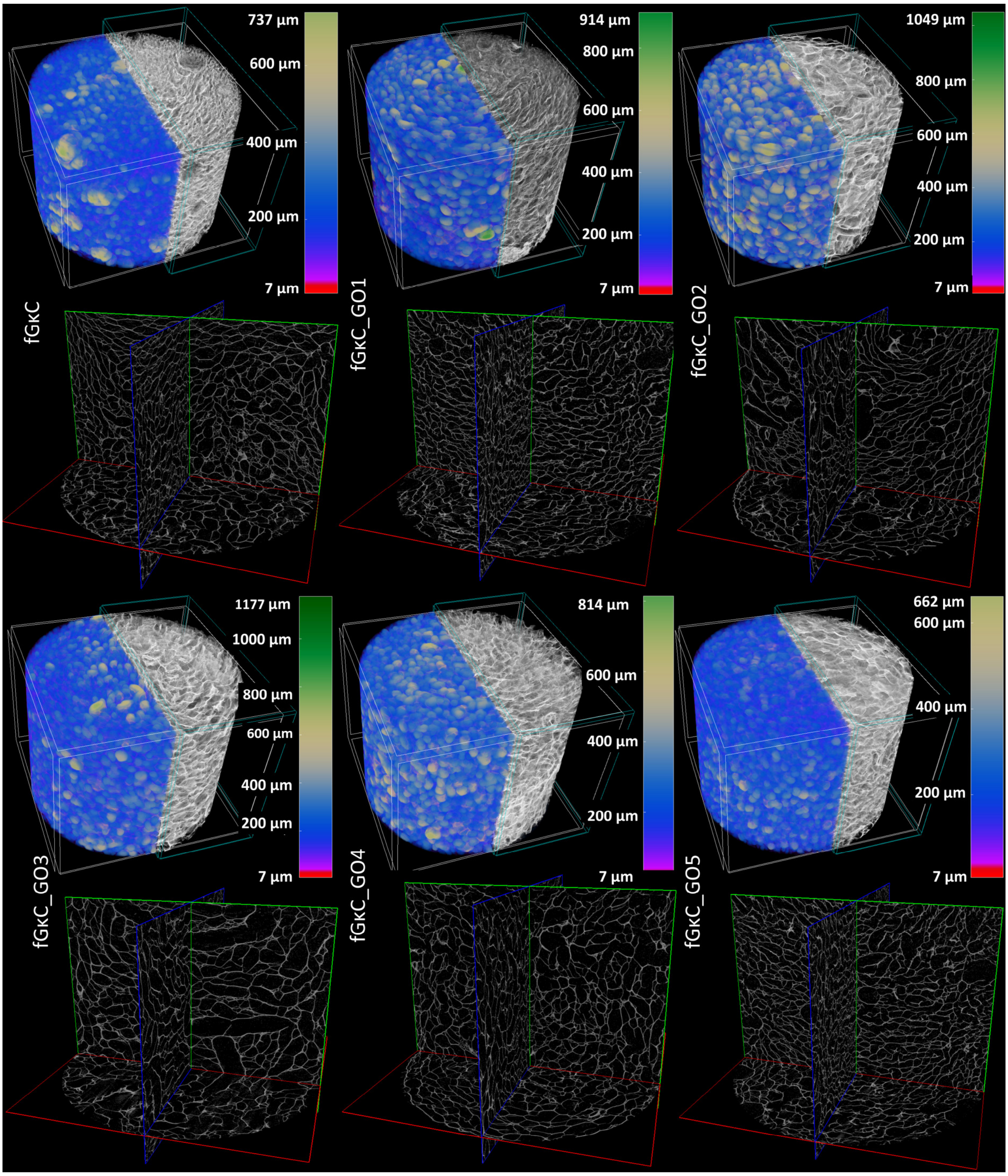

Figure S1. Captures of reconstructed tomograms illustrated in CTVox (color figures) coupling the reconstructed pores as objects over the porous structures of the samples and representations of the intersections of the XY/XZ/ZY planes extracted from DataViewer (black and white subdivisions).
